# Exosome circATP8A1 induces macrophage M2 polarization by regulating the miR-1-3p/STAT6 axis to promote gastric cancer progression

**DOI:** 10.1186/s12943-024-01966-4

**Published:** 2024-03-08

**Authors:** Cuncan Deng, Mingyu Huo, Hongwu Chu, Xiaomei Zhuang, Guofei Deng, Wenchao Li, Hongfa Wei, Leli Zeng, Yulong He, Huashan Liu, Jia Li, Changhua Zhang, Hengxing Chen

**Affiliations:** 1 Digestive Diseases Center, Guangdong Provincial Key Laboratory of Digestive Cancer Research, The Seventh Affiliated Hospital, Sun Yat-Sen University, Shenzhen, China; 2 The Biobank, Scientific Research Center, The Seventh Affiliated Hospital of Sun Yat-Sen University, Shenzhen, 518107 Guangdong People’s Republic of China; 3 Department of General Surgery (Colorectal Surgery), The Sixth Affiliated Hospital, Sun Yat-Sen University, Guangzhou, China; 4 Clinical Research Center, The Seventh Affiliated Hospital, Sun Yat-sen University, Shenzhen, China

**Keywords:** Gastric cancer, Exosomes, Macrophages, M2 polarization, STAT6

## Abstract

Circular RNAs (circRNAs) play important roles in gastric cancer progression but the regulatory role of circRNAs in controlling macrophage function remains elusive. Exosomes serve as cargo for circRNAs and play a crucial role as mediators in facilitating communication between cancer cells and the tumor microenvironment. In this study, we found that circATP8A1, a previously unreported circular RNA, is highly expressed in both gastric cancer tissues and exosomes derived from plasma. Increased circATP8A1 was associated with advanced TNM stage and worse prognosis in patients with gastric cancer. We showed that  the circATP8A1 knockdown significantly inhibited gastric cancer proliferation and invasion in vitro and in vivo. Functionally, exosome circATP8A1 induced the M2 polarization of macrophages through the STAT6 pathway instead of the STAT3 pathway. Mechanistically, circATP8A1 was shown to activate the STAT6 pathway through competitive binding to miR-1-3p, as confirmed by Fluorescence In Situ Hybridization (FISH), RNA immunoprecipitation, RNA pulldown, and Luciferase reporter assays. The reversal of circATP8A1-induced STAT6 pathway activation and macrophage polarization was observed upon blocking miR-1-3p. Macrophages treated with exosomes from gastric cancer cells overexpressing circATP8A1 were able to promote gastric cancer migration, while knockdown of circATP8A1 reversed these effects in vivo. In summary, exosome-derived circATP8A1 from gastric cancer cells induce macrophages M2 polarization via the circATP8A1/miR-1-3p/STAT6 axis, and tumor progression. Our results highlight circATP8A1 as a potential prognostic biomarker and therapeutic target in gastric cancer.

**Supplementary Information:**

The online version contains supplementary material available at 10.1186/s12943-024-01966-4.

## Introduction

Gastric cancer (GC) is the world's fifth most common cancer and is the third leading cause of cancer-related deaths globally [1, 2]. Gastric cancer frequently manifests insidiously with atypical or even no discernible symptoms. The vast majority of gastric cancer patients are diagnosed at an advanced stage, by which time lymph node involvement and distant organ metastasis are common [3, 4]. Consequently, the prognosis for patients with advanced-stage gastric cancer remains poor and identifying mechanisms facilitating local infiltration and distant metastasis is essential for early diagnosis and treatment.

Circular RNAs are non-coding RNAs that are abundant in mammalian cells and consist of diverse RNA sequences and structural domains. CircRNAs form a covalently closed loop without a 5' cap or 3' tail [5]. CircRNAs exhibit tissue- or stage-specific expression and are more ribonuclease-resistant than linear mRNAs, ensuring greater stability. While primarily localized in the cytoplasm, some circRNAs are remarkably abundant in the nucleus [6]. CircRNAs function as competitive endogenous RNAs (ceRNAs) to modulate miRNA target expression, interact with proteins, regulate alternative splicing, control parental gene transcription, and can be translated into proteins or polypeptides [7]. Chen et al. found that circPVT1 is upregulated in gastric cancer tissues and cell lines and competitively binds to miR-125, thereby promoting gastric cancer cell proliferation and serving as a prognostic marker for gastric cancer [8]. Circular RNA circNRIP1 acts as a sponge for microRNA-149-5p and promotes gastric cancer progression through the AKT1/mTOR pathway [9]. CircRNAs play a pivotal role in gastric cancer progression and have potential as prognostic markers [10, 11].

Exosomes are nano-sized particles produced and secreted by cancer cells, with diameters ranging from 20 to 150 nm. Exosomes serve as crucial mediators of intercellular communication by transporting a repertoire of molecules, including circular RNAs, microRNAs, messenger RNAs, proteins, and lipids, to target cells to modulate various biological processes [12]. Recent evidence suggests that exosomes play an important role in the progression of gastric cancer [13]. Studies have revealed notably distinct exosomal circRNA expression profiles in the plasma of individuals with gastric cancer compared to normal subjects [14]. For instance, exosome circSHKBP1 promotes gastric cancer progression via the miR-582-3p/HUR/VEGF pathway, positioning circSHKBP1 as a potential circulating biomarker for gastric cancer diagnosis [15].

The tumor microenvironment (TME) encompasses the entirety of tumor cells, non-tumor components, and their associated metabolites within a specific spatial context. It includes tumor cells, tumor-associated immune cells, endothelial cells, extracellular matrix, and fibroblasts [16, 17]. Tumor-associated macrophages (TAMS) are a versatile heterogeneous population of cells in the tumor microenvironment, that can make up to 50% of some solid tumors [18]. In the gastric cancer microenvironment, TAMs predominantly exhibit a pro-carcinogenic M2 phenotype, which is a key factor in gastric cancer progression [19]. Exosomes serve as crucial mediators for material transfer and information exchange between tumors and their microenvironment [20]. However, the impact of gastric cancer-derived exosome circRNAs on TAMs is not yet fully understood.

M2-type macrophages in the tumor microenvironment are closely associated with the gastric cancer progression. The STAT pathway is a more central signaling pathway in macrophage polarization. The balance between STAT1 and STAT3/STAT6 activation regulates macrophage polarisation and activity. Activation of NF-κB and STAT1 pathways induced macrophage M1 polarization, exerting inflammatory functions and cytotoxic effects, whereas activation of STAT3 and STAT6 pathways mediated macrophage M2 polarization, suppressing tumour immunity and promoting gastric cancer progression [21]. M2-type markers were increased in macrophages overexpressing STAT6 [22], whereas STAT6 knockdown reduced M2-type marker expression [23]. miRNAs was involved in the regulation of STAT6 signaling pathway in macrophages [24].

Here, we identified differential circRNAs present in gastric cancer plasma exosomes by circRNA microarray. Our findings indicate that a novel circular RNA, circATP8A1, has an oncogenic role in the progression of gastric cancer. Through both in vitro and in vivo experiments, we demonstrated that exosomal circATP8A1 induces M2 polarization in macrophages by acting as a sponge for miR-1-3p, thereby regulating the STAT6 pathway and ultimately promoting the progression of gastric cancer.

## Methods

### Human tissue and follow-up data

The collection and processing of clinical samples for this research project were conducted with the approval granted by the Academic Ethics Review Committee of Sun Yat-sen University (No. KY-2022–051-02). These samples were obtained from patients who had been admitted to the Seventh Affiliated Hospital of Sun Yat-sen University and had received a diagnosis of gastric cancer based on pathological examination. In the case of both normal subjects and gastric cancer patients, blood samples were gathered only after obtaining informed consent from the individuals, who voluntarily signed informed consent forms.

### Cell lines culture

Gastric cancer cells (AGS, SGC-7901, HGC-27, and MKN-45) and GES-1 cells were obtained from the Shanghai Institute of Cell Biology, Chinese Academy of Sciences. Each cell line was accompanied by STR identification reports to ensure their authenticity. The cells employed in the experiments were at a passage number below 25 and were freshly retrieved from cryopreservation. Gastric cancer cells were maintained in complete growth media containing 10% FBS (Bio-channel) and 1% penicillin–streptomycin at 37 °C supplemented with 5% CO2. Cells in the logarithmic growth phase were used for in vitro experiments.

### Plasma exosome circRNA microarray analysis

Plasma exosomes were obtained through ultracentrifugation of samples isolated from three healthy individuals and three patients with gastric cancer and processed by Heyuan Biotechnology for circRNA microarray analysis. Differential circRNAs in this study were identified with follow criteria: a *p*-value less than 0.05 and a minimum fold change of 1.5. Statistical methods such as t-tests or ANOVA were employed to identify circRNAs that exhibited significant differences based on experimental type distinctions.

### RNA and gDNA extraction

Total RNA was isolated using a TRIzol reagent (Invitrogen, USA). Relative RNA quantities were determined using the standard 2-ΔΔCt method. Genomic DNA was isolated from AGS and SGC-7901 gastric cancer cells using the Dzup (Plant) Genomic DNA Isolation Reagent (Sangon Biotech, Shanghai, China), following the manufacturer's instructions. The RNA extracted from AGS and MKN-45 cells underwent a fifteen-minute RNase R treatment at 37 °C, followed by a ten-minute incubation at 70 °C to deactivate RNase R. Subsequently, circATP8A1 and ATP8A1 mRNA stability were determined through qRT-PCR analysis.

### cDNA synthesis and qPCR and digital PCR to detect circular RNAs

Complementary DNA (cDNA) for mRNA was synthesized using the PrimeScript RT Master Mix kit. Reverse transcription for circRNA was performed using the riboSCRIPT Reverse Transcription Kit (Ribobio, Guangzhou, China). For miRNAs, reverse transcription was conducted using the miRNA First Strand cDNA Synthesis (Tailing Reaction) kit (Sangon Biotech, Shanghai, China). qRT-PCR was conducted using SYBR Premix Ex Taq I. β-Actin was utilized as the internal control for mRNA analysis, whereas U6 served as the internal control for miRNA analysis.

### Overexpression and knockdown of circATP8A1

To achieve the knockdown of circATP8A1, three siRNAs were purchased from Ribobio and transfected into AGS and MKN-45 cells using Lipo6000 (Biotool) transfection reagent. The two siRNAs exhibiting the most effective knockdown were chosen for the construction of shRNA. These shRNAs were cloned into the pLKO.1 vector. For the development of the overexpression plasmid, the circATP8A1 sequence was cloned into the pLO5-ciR lentiviral vector (Geneseed Biotech Co., Ltd). Lentiviruses were obtained by cotransfecting shRNA vectors along with packaging plasmids pMD2G and pSPAX2 (kindly provided by Songyang Zhou, Baylor College of Medicine) into HEK-293 T cells. Forty-eight hours after transfection, the supernatant was collected for infection of cancer cells. Infected cells were selected with media containing puromycin (2 ug/ml) for 72 h.

### Cell migration and invasion assays

Gastric cancer cells (AGS, SGC-7901, and MKN-45) were seeded onto Transwell inserts with pore sizes of 8 µm or 12 µm. The upper chamber was filled with serum-free corresponding culture medium, while the lower chamber contained culture medium supplemented with 10%-20% FBS. Following an incubation period ranging from 24 to 96 h, the cells were fixed using 4% paraformaldehyde and subsequently stained with 0.1% crystal violet. The stained cells were captured using a Shunyu optical microscope and quantified in five randomly selected fields, with mean values calculated for analysis.

### Cell Counting Kit-8 (CCK8) assays

For the CCK-8 assay, AGS, SGC-7901, and MKN-45 cells were seeded into 96-well plates at a density of 1,200 cells per well. The optical density at 450 nm (OD450) of each well was measured at 0, 24, 48, 72, and 96 h after seeding, following the manufacturer's instructions.

### Colony-formation assays

For the colony-formation assay, transfected cells were seeded into 6-well plates at a density of 1000 cells per well and cultured for 10–20 days in the aforementioned medium. The resulting colonies were fixed using 4% paraformaldehyde and subsequently stained with 0.1% crystal violet. Following staining, the colonies were imaged and counted.

### Isolation and identification of exosomes

**Fig. 1:**
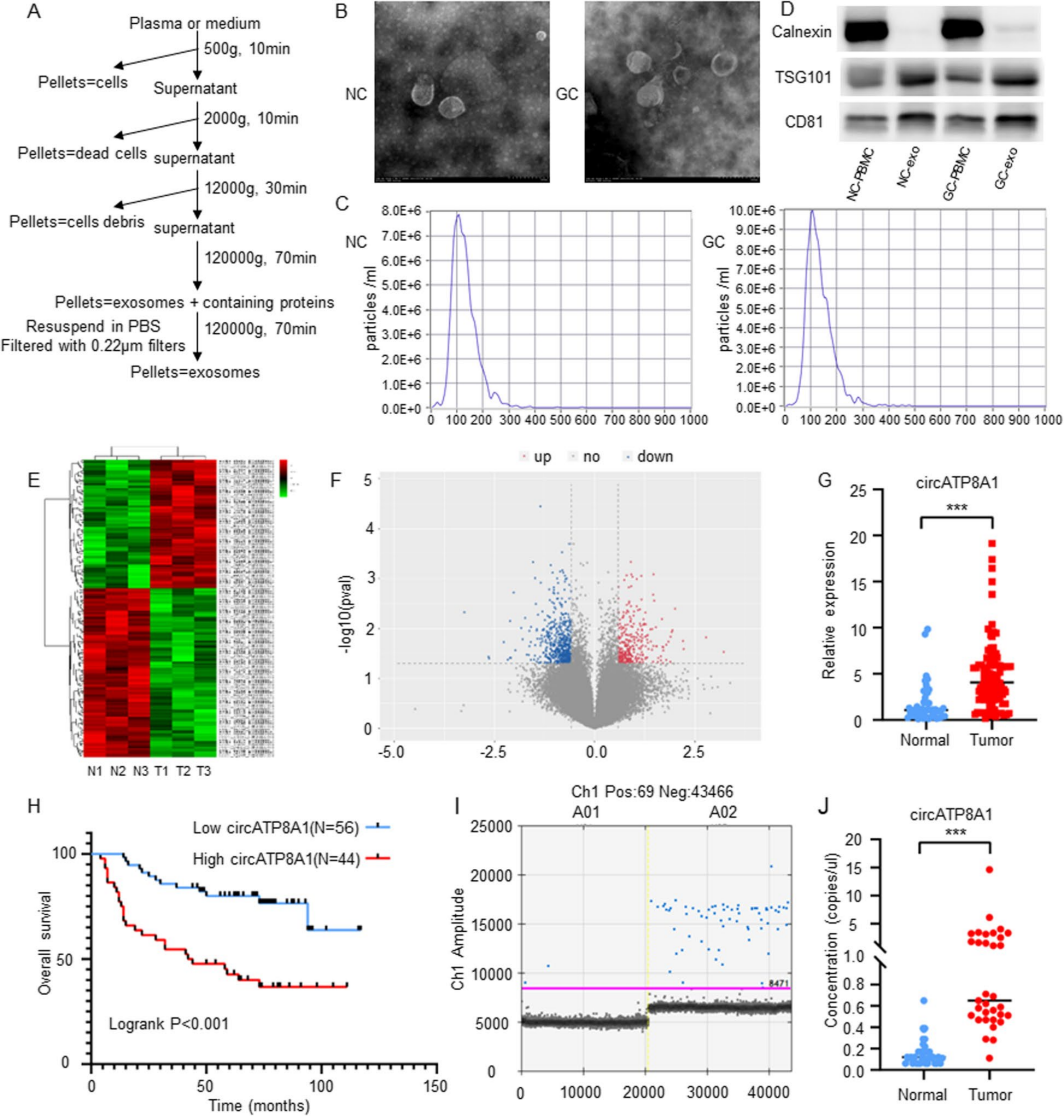
Identifying and validating a novel oncogenic exosome circATP8A1 in Gastric Cancer. **A** Workflow diagram for isolating exosomes from the plasma of healthy individuals and gastric cancer patients. **B** TEM images of exosomes in the plasma of normal individuals and gastric cancer patients. **C** Nanoparticle tracking analysis (NTA) of exosomes in the plasma of normal individuals and gastric cancer patients. **D** Western Blot analysis of exosome markers CD81, TSG101. Calnexin as a negative control. **E** Heatmap of differential exosome circRNA expression in plasma between healthy individuals and gastric cancer patients. **F** Volcanic map of differential exosome circRNA expression in plasma between healthy individuals and gastric cancer patients. **G** The expression of circATP8A1 in gastric cancer tumor tissues (*N* = 100) and normal tissue (*N* = 42) was verified by qPCR. **H** Kaplan–Meier analysis of overall survival of circATP8A1 in our cohort. **I** & **J** The expression of circATP8A1 in gastric cancer tumor tissues and healthy individuals (Normal) was verified by digital PCR. Gastric cancer tumor tissues (*N* = 36), Normal (*N* = 36). Data are presented as mean ± SD. *P* value is determined by nonparametric test for G and J, log-rank text for H. *** P < 0.001

Plasma exosomes were isolated via ultracentrifugation [25], and their concentration was quantified using the BCA assay (Fig. 1).

The morphology of the exosomes' particles was characterized using transmission electron microscopy (JEM-1400/JEM-1400 PLUS, Japan). Western blot analysis was employed to determine exosome protein markers. Nanoparticle tracking analysis (NTA) of the exosomes was conducted by Bioyard Biotechnology Development Co., Ltd (Guangzhou).

### THP1 cell culture and M0 macrophage induction

The THP-1 cells were cultured in RPMI Medium 1640, supplemented with 10% FBS (Nanjing Ozfan), 2.5 g/L D-Glucose, and 10 mM Hepes.Na, 1% PS, and 0.05 mM β-mercaptoethanol. To induce differentiation of THP-1 cells, they were incubated in the aforementioned medium at 37 °C in a cell culture incubator with 5% CO2. Following 24 h of induction with PMA (100 ng/ml), the THP-1 cells adhered to the culture vessel, undergoing differentiation into M0 macrophages. Treating M0 macrophages with exosomes derived from GC cells overexpressing circATP8A1. Exosomes from conditioned media of both circATP8A1 overexpressing and control cells were separately harvested using ultracentrifugation. THP-1 cells were then induced into M0-type macrophages through PMA stimulation, followed by treatment with 20 μg/mL of extracellular vesicles (EVs). After 48 h, RNA and proteins were collected for subsequent experiments or co-culturing with gastric cancer cells.

### Exosome uptake assay

PKH67, a Green fluorescent dye (Sigma-Aldrich, USA), was used to label exosomes to trace their uptake by macrophages. Subsequently, we examined the samples using confocal microscopy at both 2 and 6 h following the PKH67 treatment.

### Immunofluorescence and immunohistochemical stainings

Cells grown on glass coverslips were fixed with 4% paraformaldehyde for 20 min, followed by preextraction with 0.5% Triton X-100 for 8 min at room temperature (RT). Slides were incubated in a blocking solution (5% bovine serum albumin (BSA)) for 1 h. and incubated with the indicated antibodies at 4 °C overnight, followed by staining with secondary antibodies at RT for 1 h. After staining with DAPI, the samples were analyzed by confocal imaging using a Zeiss LSM 800.

### Single-cell sequencing data analysis of gastric cancer

Single-cell RNA-seq data were retrieved from the GEO database (GSE183904) to cluster cells according to different markers. The macrophages were clustered according to the M1 and M2 markers. Relative genes in M1 and M2 macrophages were explored.

### Induction of M2 macrophage polarization through IL-4 stimulation

THP1 cells were initially stimulated with 100 ng/mL of PMA for 24 h, leading to their differentiation into M0 macrophages. Then, the cells were subjected to treatment with IL-4 at a concentration of 50 ng/mL for an additional 24 h, inducing M2 polarization of the macrophages. Subsequent analyses were conducted after 48 h.

### Bioinformatics analysis for constructing a circRNA ceRNA network

We conducted predictions to identify miRNAs with potential binding sites on circATP8A1 using the ENCORI database (https://rnasysu.com/encori/). Similarly, predictions for miRNAs capable of binding to STAT6 and its upstream were also performed using the ENCORI database. The intersection of these two sets of miRNAs revealed a shared set of miRNAs. Utilizing bioinformatics analysis, we constructed a putative ceRNA network, establishing a computational framework to guide subsequent experimental investigations.

### Fluorescence in situ hybridization (FISH)

The FISH experiment was carried out to detect the subcellular localization of circATP8A1 and miR-1-3p. A FISH kit (c10910, RiboBio, Guangzhou, China) was employed, following the manufacturer's instructions. The hybridization process involved the utilization of Cy3-labeled circATP8A1 probes and FAM-labeled miR-1-3p probes from Sangon Biotech, Shanghai, China. DAPI staining solution, which contained a fluorescence quencher (Abcam), was applied to the slides, and coverslips were placed over them for sealing. Finally, the results were analyzed through confocal microscopy.

### RNA immunoprecipitation

The RIP experiment was conducted using anti-Ago2 and anti-IgG antibodies. The RIP experiment was performed using the Magna RIP RNA-binding protein immunoprecipitation kit (Millipore, Merck, Germany) following the manufacturer's instructions. Enrichment values were normalized against the background RIP levels detected using the IgG isotype control.

### Biotin-labeled probe RNA pull-down experiment

Biotinylated miR-1-3p and scrambled negative control miRNA were synthesized (RiboBio, China). Biotinylated miRNAs were transfected into cells using Lipofectamine 6000 (Invitrogen, USA), then cell lysates were collected 24 h after transfection and then fixed with 3% paraformaldehyde for 30 min, followed by incubation with 1.25 M glycine for 5 min at RT. Centrifuge 1500 g-2000 g for 5 min, discard the supernatant, add PBS to the suspended cells, and count. Then cell lysates were collected 48 h after transfection and incubated with streptavidin magnetic beads (Invitrogen, USA) at RT for 2 h. After centrifuging to wash the beads, pulled-down miRNAs were extracted using Trizol (Invitrogen, USA) and subjected to qRT-PCR. The primers for qRT-PCR were showed in Supplementary data Table S1.

### Luciferase reporter assay

The wild-type or mutant variants of circATP8A1 or STAT6 were amplified and individually cloned into the pmirGLO vector. Subsequently, HEK-293 T cells were seeded into a 96-well plate and co-transfected with either the wild-type or mutant luciferase plasmids, in combination with miR-1-3p or a control miRNA.

### ELISA analysis of supernatants from macrophages after exosome treatment

The IL-10, TGFβ, and CXCL1 concentration in cell culture medium was measured by ELISA using the Quantikine human ELISA kit (Wuhan Fine Biotech Co., Ltd) according to the manufacturer’s instructions.

### Coculturing macrophages treated with exosomes with gastric cancer cells

THP1 cells were seeded in a 24-well plate and subjected to PMA treatment to induce their differentiation into M0 macrophages. Subsequently, they were separately exposed to exosomes derived from cells overexpressing circATP8A1 and control cells. After 12 h, the culture medium was replaced with fresh medium, and a small chamber containing SGC-7901 cells was positioned above the 24-well plate. The upper chamber contained a serum-free medium, while the lower chamber was filled with a medium containing 10% serum. Following 24 h of co-culture, the cells were fixed with paraformaldehyde, stained with crystal violet, and then photographed.

### Animal experiments

All animal care and experimental protocols were approved by the Institutional Animal Care and Use Committee (IACUC) of Sun Yat-sen University (No. SYSU-IACUC-2023-B0965) by the standards of the National Institutes of Health. BALB/c nude mice (3 ~ 4 weeks old) were purchased from the Guangdong Vital River Laboratory Animal Technology Co., Ltd (China). The MKN-45 cell line, stably transfected with the control vector and circATP8A1 shRNA, was utilized for both animal experiments and exosome isolation.

First, to investigate the effect of circATP8A1 on gastric cancer, all mice were divided into two groups (*n* = 6), including the control group (shNC) and circATP8A1 knockdown group(sh1-circATP8A1). MKN-45 cells (5 × 10^6^) transfected with the control vector or circATP8A1 shRNA were inoculated subcutaneously on the left side of the body. Second, to investigate the effect of exosomes and exosome circATP8A1 on gastric cancer, all mice were divided into three groups (*n* = 6), including the PBS group (PBS), the knockdown-control exosomes group (shNC-exo), and the circATP8A1 knockdown  exosomes group (sh1-circATP8A1-exo). Each nude mouse was subcutaneously implanted with approximately 6 × 10^6^ MKN45 cells along with approximately 1.2 × 10^6^ macrophages. 7–9 days after cell injection, when tumors were formed, the PBS group, the shNC-exo group, and the sh1-circATP8A1-exo group were injected intratumourally with 100 µl PBS, 60 µg/100 µl exosomes derived from shNC and sh1-circATP8A1 MKN45 cells, respectively. Exosome injections were administered every 3 days for a total of three times. Tumor volumes were measured every 3 days. All mice were sacrificed after 30 days and the subcutaneous tumor was dissected, collected, and weighed. Hematoxylin and eosin (H&E) staining was performed, followed by immunohistochemical analysis to evaluate the expression of the proliferation marker Ki67.

### Statistical analysis

All statistical analyses were performed with GraphPad Prism 7.0 (GraphPad). Values were obtained from at least three independent experiments, using three technical replicates per condition, unless otherwise indicated in the figure legend. No animals or tumor samples were excluded from data analyses. Student’s t-test, two-sided, unpaired, two-tailed, two-way, or one-way analysis of variance (ANOVA) was used to analyze data as indicated. The Kaplan–Meier method was used to calculate the cumulative overall survival data, and the log-rank test was used for analysis.

More detailed procedures were provided in Supplemental Methods.

## Results

### Identification and validation of a novel oncogenic exosome circATP8A1 in gastric cancer

Exosomes were isolated from the plasma of both healthy individuals and GC patients (Fig. 1A, B). Nanoparticle Tracking Analysis (NTA) showed that the diameter of exosomes from normal plasma was 116.8 nm ± 54.1 nm, with a concentration of 7.7 × 10^9^ particles/ml (Fig. 1C), consistent with previously reported characteristics of exosomes [26]. The diameter of exosomes from gastric cancer plasma was 110.6 nm ± 55.7 nm, with the same concentration of 7.7 × 10^9^ particles/ml (Fig. 1C). Western blot (WB) assays confirmed high expression levels of the exosome markers CD81 and TSG101 in both healthy and GC plasma exosomes in contrast to the control PBMCs (Fig. 1D).

To identify the differential presence of circRNAs in gastric cancer plasma exosomes, we performed circRNA microarrays on plasma exosomes from three normal subjects and three gastric cancer patients. Differentially expressed circRNAs were identified with a log2 fold change > 1 or < -1 and *P* < 0.05, visualized in the heatmap (Fig. 1E) and volcano plot (Fig. 1F). The qPCR results showed that circATP8A1 expression was significantly increased in gastric cancer tissue (*P* < 0.01, Fig. 1G). Importantly, higher circATP8A1 expression in gastric cancer tissue was significantly correlated with a poorer prognosis for GC patients (Fig. 1H) and correlation analysis revealed that circATP8A1 expression was associated with clinicopathological features including T stage, N stage, M stage, and TNM stage (*P* < 0.05, Table 1). Besides, the association of circATP8A1 expression with clinicopathological features in plasma exosomes of gastric cancer patients were showed in Supplementary data Table S2. Furthermore, univariate and multivariate Cox analysis demonstrated that higher circATP8A1 expression was related to poor survival outcomes (*P* < 0.01, Table 2). To validate these data in a larger cohort, we analyzed circATP8A1 expression in plasma exosomes of 36 normal subjects and 36 gastric cancer patients by digital PCR (ddPCR). The results confirmed that the expression level of circATP8A1 in plasma exosomes of gastric cancer patients was significantly higher than that of the normal group (*P* < 0.01, Fig. 1I, J).

**Table 1:** Association of circATP8A1 expression with clinicopathological features in gastric cancer

Characteristics (*n* = 100)	CircATP8A1 expression		
	**Negative(*n* = 56)**	**Positive(*n* = 44)**	***P***
Age (years)			0.232
< 60	26	26	
≥ 60	30	18	
Gender			0.192
Male	42	27	
Female	14	17	
Weight	58.94 ± 9.81	58.13 ± 9.66	0.689
Height	165.09 ± 6.35	161.66 ± 8.53	0.06
Location			0.006
Upper third	21	14	
Middle third	2	10	
Lower third	33	17	
Whole	0	2	
T stage			0.002
T1	21	4	
T2	15	9	
T3	11	14	
T4	9	17	
N stage			0.003
N0	31	18	
N1	6	6	
N2	13	3	
N3	6	17	
M stage			0.012
Negative	52	32	
Positive	4	12	
TNM Stage			0.041
I	23	11	
II	14	11	
III	15	10	
IV	4	12	
Differentiation			0.937
Well	4	3	
Moderately	17	12	
Poorly	35	29

**Table 2:** Univariate and multivariate Cox regression analyses of clinical parameters on overall survival for 100 gastric cancer patients

Parameter	Univariate analysis		Multivariate analysis	
	HR (95% CI)	*P* value	HR (95% CI)	*P* value
Age (≥ 60 vs < 60 yr)	1.179(0.633–2.193)	0.604	1.001(0.513–1.953)	0.997
Gender (males vs females)	0.624(0.329–1.184)	0.149	1.070(0.518–2.207)	0.855
T stage (3,4 vs 1,2)	0.122(0.051–0.293)	< 0.001	0.292(0.107–0.802)	0.017
N stage (N2 ~ N3 vs N0 ~ N1)	0.164(0.082–0.327)	< 0.001	0.556(0.181–1.705)	0.305
M stage (M1 vs M0)	0.157(0.080–0.309)	< 0.001	0.536(0.229–1.256)	0.151
TNM stage (III, IV vs I, II)	0.103(0.048–0.221)	< 0.001	0.248(0.068–0.897)	0.034
circATP8A1 expression (High vs low)	0.275(0.142–0.533)	< 0.001	0.359(0.161–0.803)	0.013

*HR* Hazard ratio, *CI* Confidence interval

### Characterization of circATP8A1 in GC

CircATP8A1 (Arraystar ID: hsa_circRNA_0069616; circBase ID: hsa_circ_0069616) is back-spliced from exon3 and exon20 of the ATP8A1 gene with a length of 1558nt originating from chromosome 4 (Figure S1A). The backsplice junction site of circATP8A1 was amplified with divergent primers and subsequently verified by Sanger sequencing (Figure S1A). The qRT-PCR assay revealed that circATP8A1 expression was elevated in the gastric cancer cell lines AGS and MKN-45 compared with GES-1 (Figure S1B). After treating AGS cells with Actinomycin D, it was observed that the circular circATP8A1 has a longer half-life compared to the linear mRNA ATP8A1. Approximately 80% of the circular form circATP8A1 remains after 24 h, while only about 20% of the linear ATP8A1 mRNA is left (Figure S1C). As expected RNase R digestion experiments showed that circular circATP8A1 was more resilient to degradation when compared to linear mRNA ATP8A1 in AGS and MKN-45 cells (Figure S1D, S1E). Two pairs of designed divergent and convergent primers were used to amplify circATP8A1 and linear mRNA ATP8A1 respectively. Agarose gel electrophoresis analysis demonstrated that the linear form of ATP8A1 can be amplified from both cDNA and gDNA, whereas circATP8A1 is only amplifiable from cDNA (Figure S1F).

### CircATP8A1 promotes proliferation and migration in gastric cancer

**Fig. 2:**
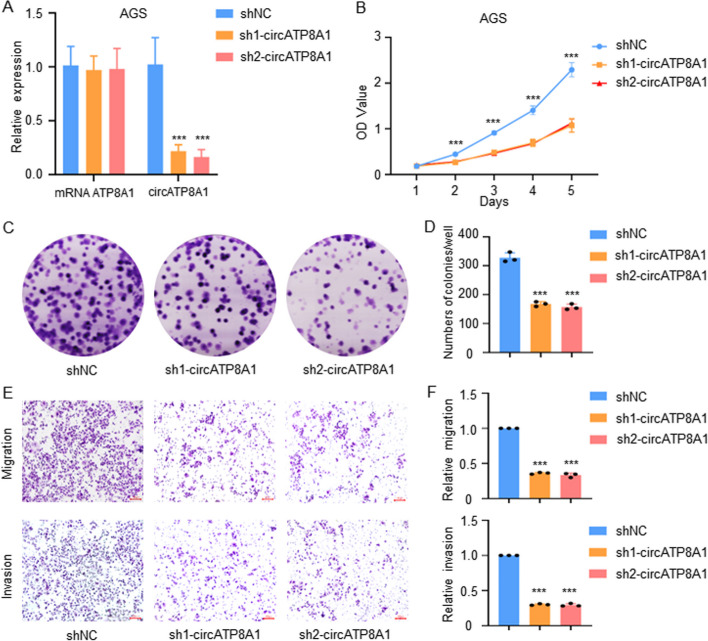
CircATP8A1 knockdown reduces proliferation and migration in AGS cells. **A** The relative expression levels of circATP8A1 and linear ATP8A1 after circATP8A1 knockout in AGS cells were detected by qRT-PCR. **B** The proliferative ability of AGS cells after circATP8A1 knockdown was detected by CCK8 assay. **C** & **D** Representative images of clone formation and statistics of colony counts in AGS cells with circATP8A1 knockdown. **E** & **F** Microscopic images and quantification of the migration and invasion of AGS cells as described above. *P* value is determined by t-test for A, B, D, and F. *** P < 0.001. shNC, the knockdown-control group; sh1-circATP8A1 and sh2-circATP8A1, circATP8A1 knockdown group1 and group2

To explore the biological role of circATP8A1 in gastric cancer, stable knockdown and overexpression of circATP8A1 were investigated. Based on the qPCR results of gastric cancer cell lines, AGS and MKN-45 cell lines with high expression of circATP8A1 were selected to construct stable lines of knockdown circATP8A1. In circATP8A1 knockdown AGS cell lines, qRT-PCR confirmed reduced circATP8A1 expression, while linear ATP8A1 mRNA was not significantly altered (Fig. 2A). Following the downregulation of circATP8A1, CCK-8 assay results revealed a notable decrease in the proliferative capacity (Fig. 2B) and the formation of plate colonies in AGS cells (Fig. 2C, D). To explore the impact of suppressing circATP8A1 on the remote metastasis capacity of AGS cells, transwell experiments were conducted. The results indicated that AGS cells with knockdown of circATP8A1 had significantly reduced migration and invasion ability (Fig. 2E, F). Similar results were observed in the knockdown of circATP8A1 in MKN-45 cells (Figure S2).

Additionally, the function of circATP8A1 overexpression was tested in SGC-7901. The results showed that overexpression of circATP8A1 (Figure S3A) could promote malignancy in gastric cancer by increased growth (Figure S3B), plate clone formation (Figure S3C, D), migration and invasion ability (Figure S3E, F).

### Exosome circATP8A1 promotes the polarization of M0 macrophages towards M2

**Fig. 3:**
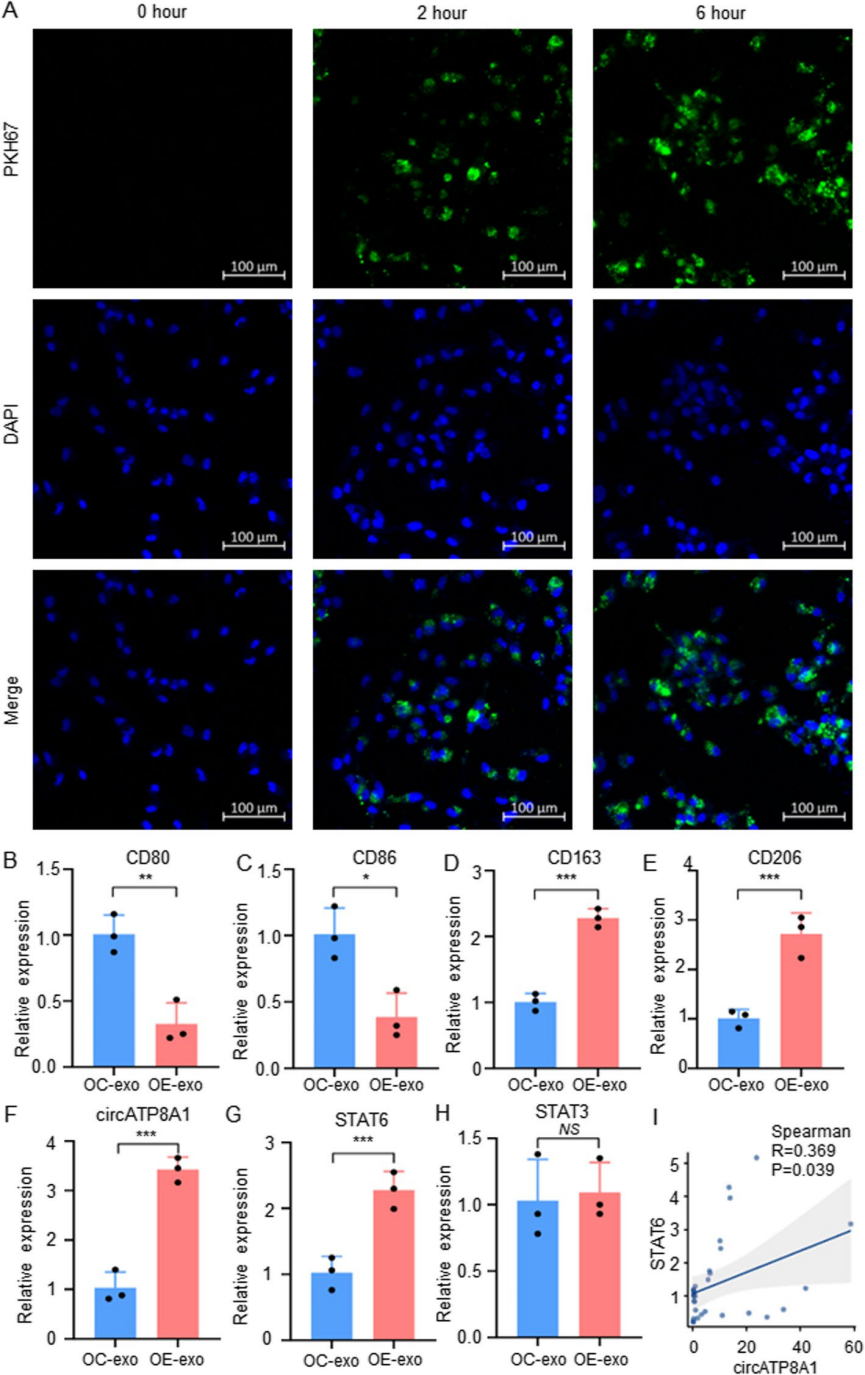
Exosome circATP8A1 promotes the polarization of M0 macrophages towards M2. **A** IF staining was performed with PKH67 and DAPI at 2 h and 6 h following exosome treatment as indicated. **B** & **C** M1-phenotype polarization markers CD80 and CD86 were detected by qPCR. **D** & **E** M2-phenotype polarization markers CD163 and CD206 were detected by qPCR. **F**–**H** CircATP8A1, STAT6, and STAT3 were detected by qPCR. **I** CircATP8A1 express was positively correlative with STAT6 in our patient cohort (*n* = 32). *P* value is determined by the t-test for B-H. * *P* < 0.05, ** *P* < 0.01, *** *P* < 0.001. *NS*, not significant. OC-exo, exsomes from overexpression control cells OE-exo, exosomes from circATP8A1 overexpression cells

To evaluate the influence of exosome circATP8A1 on macrophages, exosomes from circATP8A1-overexpressing SGC-7901 cells were collected to treat M0 macrophages. Exosomes from gastric cancer cells were collected and identified with electron microscopy, nanoparticle tracking analysis (NTA), and WB (Figure S4). Exosomes were stained with PKH67 and the uptake of exosomes by macrophages was assessed using fluorescence detection. Results showed that 2 h after being treated, exosomes began to enter macrophages (green fluorescence), and by 6 h a considerable number of exosomes had entered the macrophages (Fig. 3A). The utilization of PKH67 labeling provided a direct visualization of the exosome uptake by macrophages, establishing a foundational basis for further functional implications of exosomes in macrophage physiology. qPCR data revealed that macrophages exposed to exosomes from SGC-7901 cells overexpressing circATP8A1 exhibited reduced M1 markers CD80 and CD86 (Fig. 3B, C) and elevated M2 markers CD163 and CD206 (Fig. 3D, E). The above results suggest that exosomes from SGC-7901 cells overexpressing circATP8A1 can promote the polarization of M0 macrophages towards M2.

We further investigated the specific mechanism by which gastric cancer exosomes induce M2 polarization in macrophages. STAT3 and STAT6 pathways are the core pathways for M2 polarization in macrophages. Using qPCR assay, we found that macrophages treated with exosomes overexpressing circATP8A1 had increased expression levels of circATP8A1 and STAT6 (Fig. 3F, G) compared to the control group, whereas there was no significant change in the expression level of STAT3 (Fig. 3H). Furthermore, correlation analysis of our patient data showed a positive correlation between circATP8A1 and STAT6 (Fig. 3I). These findings indicate that the STAT6 pathway, rather than STAT3, plays a role in exosome-mediated macrophage M2 polarization in SGC-7901 cells overexpressing circATP8A1.

### Elevated expression of STAT6 in gastric cancer tissues and M2-polarized macrophages

**Fig. 4:**
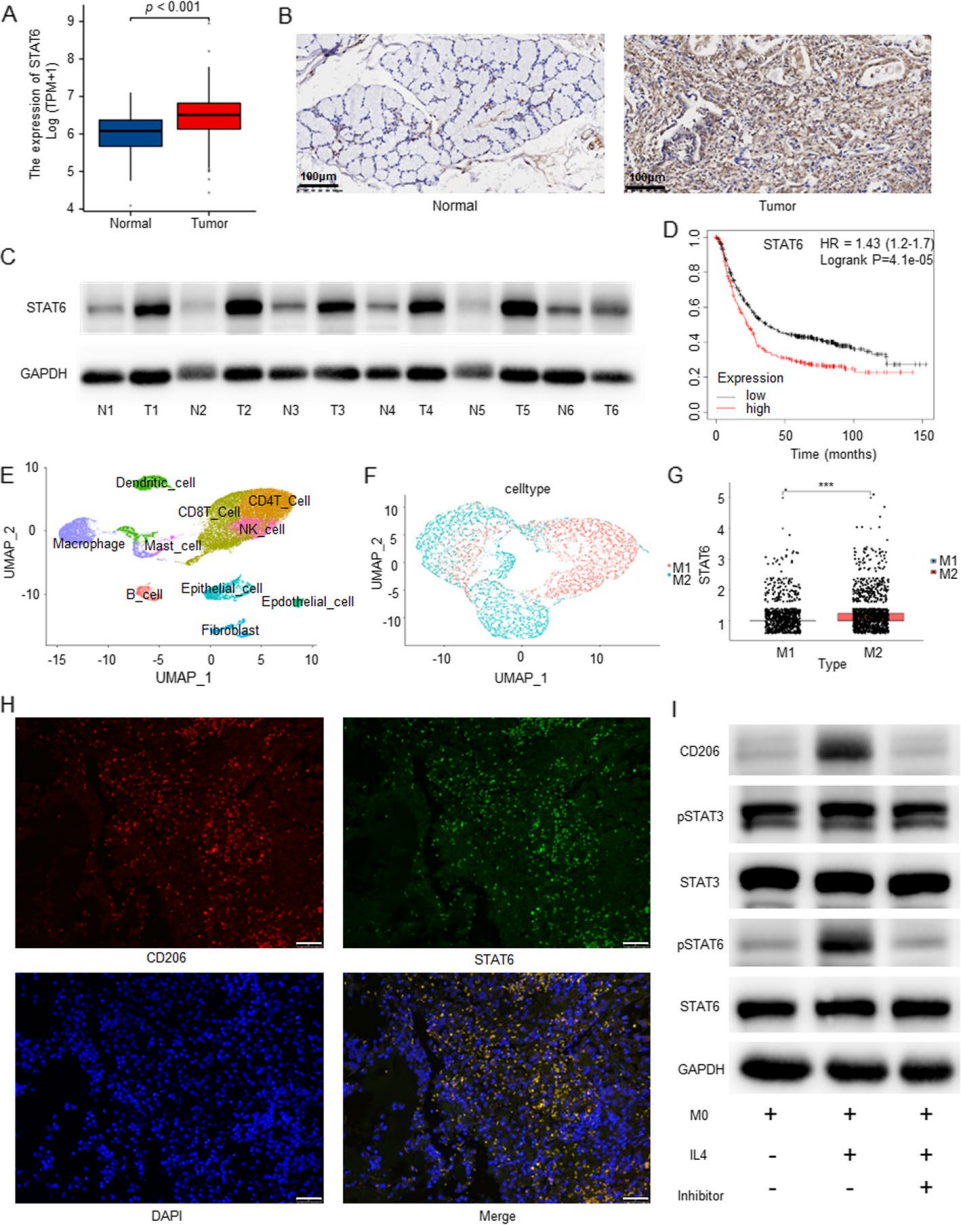
High STAT6 expression in gastric cancer tissues and M2 polarized macrophages. **A** The TCGA database showed that STAT6 was highly expressed in gastric cancer tissues compared with adjacent tissues. **B** The expression level of STAT6 in normal and gastric cancer tissues was detected by immunohistochemistry. Scale bar = 100 μm. **C** The survival analysis of STAT6 was performed using the KM plot database. **D** Western blot analysis indicated that STAT6 was highly expressed in gastric cancer tissues compared with adjacent tissues. **E** Cells were grouped based on single-cell sequencing data. **F** The macrophages were grouped according to M1 and M2 markers. **G** Single-cell sequencing data showed that STAT6 expression was higher in M2 macrophages compared with M1 macrophages. **H** IF staining was performed with anti-STAT6 and anti-CD206 in gastric cancer tissue. Scale bar = 100 μm. **I** After THP1 cells were treated with PMA, the cells were treated with IL4 or AS1517499, and the related proteins were finally detected by western blot. *** P < 0.001. N(1–6), normal tissue; T(1–6), gastric cancer tissue

The aforementioned findings imply a potential correlation between exosome-induced macrophage M2 polarization and the STAT6 pathway. Thus, we proceeded to explore the impact of STAT6 on macrophages and gastric cancer. The TCGA database showed elevated levels of STAT6 expression in gastric cancer tissues which was confirmed by our immunohistochemical results in gastric cancer samples (Fig. 4A, B). Western blot analysis indicates increased levels of STAT6 protein expression in GC tissues (Fig. 4C). High expression of STAT6 was associated with a worse prognosis through the KM plot database (Fig. 4D). These findings strongly indicate a high expression of STAT6 in gastric cancer and suggest that it may act as a pro-cancer factor.

We further employed single-cell sequencing analysis of gastric cancer to cluster cells according to different markers (Fig. 4E). Then, we proceeded to cluster the macrophages according to the M1, and M2 markers (Fig. 4F). Analysis of single-cell sequencing data revealed a marked increase in STAT6 expression levels in M2-type macrophages, indicating its involvement in macrophage M2 polarization (Fig. 4G). Subsequently, we analyzed the localization of STAT6 and the macrophage M2 polarization marker CD206 by immunofluorescence and found co-localization of STAT6 and CD206 in gastric cancer tissue (Fig. 4H), suggesting the involvement of STAT6 in macrophage polarization. We further investigated the function of the STAT6 pathway in macrophage polarization through rescue experiments. THP1 cells were treated with PMA to induce macrophage differentiation into the M0 type. Subsequently, macrophage polarization towards the M2 type was induced with IL4. Macrophages induced by IL4 exhibited markedly elevated levels of CD206 expression and pSTAT6 expression (Fig. 4I) which was reduced after treatment of STAT6 pathway inhibitors AS1517499 (Fig. 4I).

### miR-1-3p is a target of circATP8A1 in gastric cancer

**Fig. 5:**
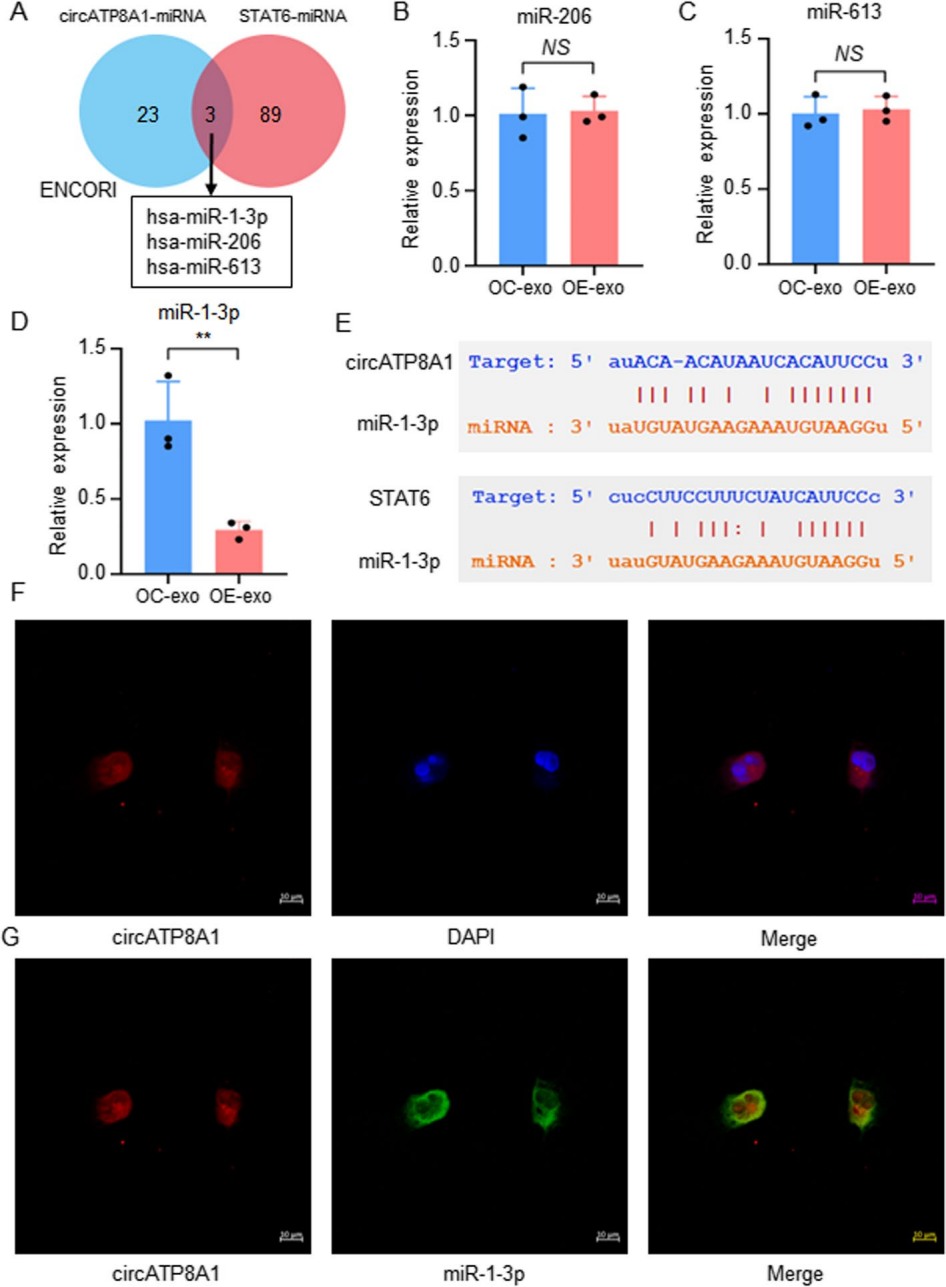
MiR-1-3p is a target of circATP8A1 in gastric cancer. **A** Three miRNAs were gained by the intersection of the predicted potential targets of circATP8A1 and STAT6 in the ENCORI database. **B**, **C**, **D** Relative expressions of miR-206, miR-613, and miR-1-3p of macrophages after treatment with exosomes overexpressing circATP8A1. **E** The binding sites between circATP8A1 and miR-1-3p, miR-1-3p, and STAT6 were predicted by the ENCORI database. **F** CircATP8A1 was mainly localized to the cytoplasm detected by FISH. Nuclear was labeled with a DAPI stain. **G** Colocalization of miR-1-3p (FAM) and circATP8A1 (Cy3) detected by FISH. * *P* < 0.05, ** *P* < 0.01, *** *P* < 0.001. *NS*, not significant

Our study identified an increase in the expression of circATP8A1 and STAT6 within macrophages post-exosome treatment of SGC-7901 cells with circATP8A1 overexpression. The core mechanism by which circRNAs exercise their functions is through ceRNA machinery. The ENCORI database was employed to narrow the miRNAs that have the potential to interact with circATP8A1 and STAT6 UTR, respectively. Three miRNA were identified from the intersection, including miR-1-3p, miR-206 and miR-613 (Fig. 5A). Macrophages treated with exosomes overexpressing circATP8A1 were detected by qPCR. The findings revealed a significant reduction in the expression of miR-1-3p (Fig. 5D), while no significant difference was found between miR-206 (Fig. 5B) or miR-613 (Fig. 5C). The predicted binding sites of circATP8A1 to miR-1-3p and miR-1-3p to STAT6 by the ENCORI database are displayed in Fig. 5E. The FISH assay was employed to analyze the subcellular localization of circATP8A1 in macrophages. The findings revealed predominant cytoplasmic localization of circATP8A1 (Fig. 5F). Moreover, colocalization of circATP8A1 and miR-1-3p in the cytoplasm was observed (Fig. 5G), providing further support for the ceRNA mechanism.

### Exosome circATP8A1 promotes macrophage M2 polarization by regulating the STAT6 pathway through competitive binding of miR-1-3p

**Fig. 6:**
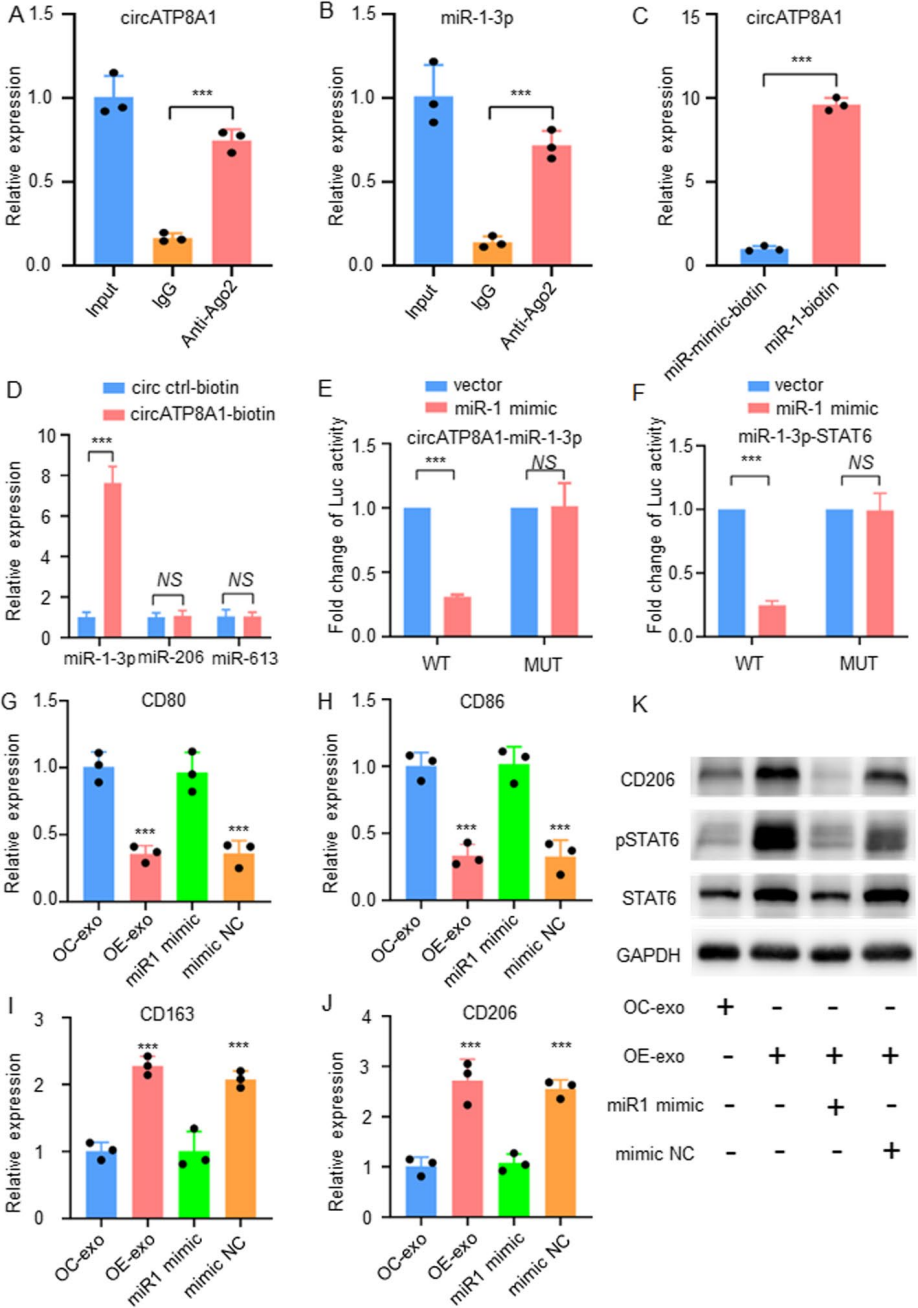
Exosome circATP8A1 promotes macrophage M2 polarization by regulating the STAT6 pathway through competitive binding of miR-1-3p. **A** RIP results showed enrichment of circATP8A1 in Ago2 immunoprecipitations (*P* < 0.05, *n* = 3). **B** RIP results showed enrichment of miR-1-3p in Ago2 immunoprecipitation (*P* < 0.05, *n* = 3). **C** The relative level of circATP8A1 was determined by qPCR after pull-down with a biotin-labeled miR-1-3p probe or oligo probe. **D** The relative level of miRNAs was determined by qPCR after pull-down with a biotin-labeled circATP8A1 probe or control probe. **E** Luciferase assay of HEK-293 T with Luc-circATP8A1-wt or Luc-circATP8A1-mut co-transfected with miR-1-3p mimic or mimic normal control. **F** Luciferase assay of HEK-293 T with Luc-STAT6-wt or Luc-STAT6-mut co-transfected with miR-1-3p mimic (miR1 mimic) or mimic normal control (mimic NC). **G**-**J** Rescue experiments were conducted to confirm the interaction between circATP8A1 and miR-1-3p. M0-type macrophages were treated with SGC7901 exosomes overexpressing circATP8A1 and further with miR1-3p mimic and mimic control. qPCR was used to detect the expression of M1 and M2 polarization markers. **K** Western blot analysis was conducted to examine the expression levels of the STAT6, pSTAT6 and CD206 in the experimental samples. * *P* < 0.05, ** *P* < 0.01, *** *P* < 0.001. *NS*, not significant

MiRNAs play a crucial role in post-transcriptional regulation, and cytoplasmic circRNAs can act as miRNA sponges to influence the translation of target genes. We first conducted RIP from macrophages using the antibody against AGO2. qPCR results showed that circATP8A1 and miR-1-3p were enriched in AGO2 immunoprecipitates (Fig. 6A, B), suggesting the formation of the RNA-induced silencing complex (RISC). Next, RNA pulldown assays showed that biotin-labeled miR-1-3p captured more circATP8A1 than a negative control probe (Fig. 6C). The biotin-labeled circATP8A1 probe was able to capture significantly more miR-1-3p, importantly there was no significant difference in the expression levels of miR-206 and miR-603 (Fig. 6D), suggesting that circATP8A1 can specifically bind to miR-1-3p. In addition, luciferase reporter assays validated that circATP8A1 binds to the wild-type miR-1-3p sequence, resulting in a more than 50% reduction in luciferase activity (Fig. 6E). The luciferase reporter assay confirmed that STAT6 binds to the wild-type miR-1-3p sequence, resulting in a more than 50% reduction in luciferase activity, but not to the mutant miR-1-3p sequence (Fig. 6F).

To further confirm the interaction between circATP8A1 and miR-1-3p, we first treated M0 macrophages with exosome from cells overexpressing circATP8A1 and then added miR-1-3p mimic and mimic control to the treatment. The qPCR results showed that the M1-type polarization markers CD80 and CD86 were significantly decreased after treatment with exosomes overexpressing circATP8A1, and the M2-type polarization markers CD163 and CD206 were significantly increased (Fig. 6G-J). Rescue experiments further confirmed that the expression levels of CD163 and CD206 were reduced after treatment with miR1 mimic. (Fig. 6G-J). The WB results further verified that the M2-type polarization marker CD206 was significantly increased after treatment of M0-type macrophages with exosomes overexpressing circATP8A1. MiR-1-3p mimic treatment can reverse exosome-induced macrophage M2 polarization and activation of the STAT6 pathway (Fig. 6K). In conclusion, these results confirmed that exosome circATP8A1 can promote macrophage M2 polarization through the activation of the STAT6 pathway and that this effect is mainly achieved through the competitive binding of miR-1-3p.

### Macrophages treated with exosomes derived from circATP8A1-overexpressing SGC-7901 cells promote the migration of GC cells

**Fig. 7:**
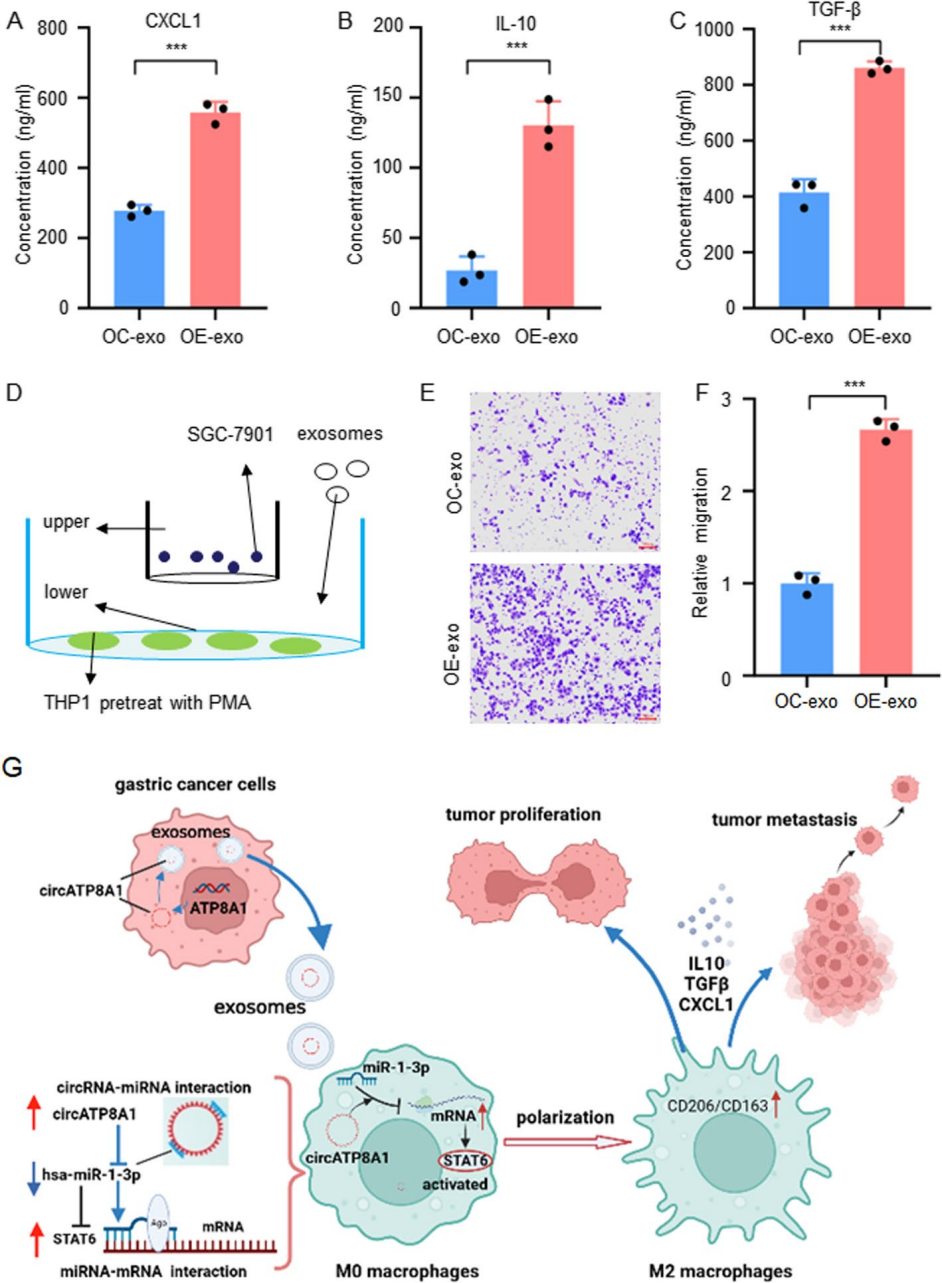
Co-culture of macrophages treated with extracellular vesicles and gastric cancer cells. **A**-**C** CXCL1, IL-10, and TGF-β levels in the supernatant of macrophages treated with exosomes from SGC-7901 cells overexpressing circATP8A1 were measured by ELISA. **D** Co-culture of macrophages treated with exosomes overexpressing circATP8A1 and SGC-7901 cells. **E**–**F** Cell migration assay of SGC-7901 after cocultured with macrophages treated with exosomes overexpressing circATP8A1. Scare bars = 100 mm. **G** Model of the exosome circATP8A1-mediated ontogenetic effect in gastric cancer. * *P* < 0.05, ** *P* < 0.01, *** *P* < 0.001. *NS*, not significant. OC-exo, exsomes from overexpression control cells; OE-exo, exosomes from cells overexpressing circATP8A1

The ELISA assay shows notably elevated levels of CXCL1, IL-10, and TGF-β expression in the macrophage supernatants after being treated with exosomes derived from circATP8A1-overexpressing SGC-7901 cells (Fig. 7A-C). We further co-cultured exosome-treated macrophages with wild-type SGC-7901 cells (Fig. 7D). Macrophages that received exosomes from SGC-7901 cells overexpressing circATP8A1 were found to promote the proliferation of gastric cancer cells (Fig. 7E, F). This could be due to the secretion of different pro-tumor cytokines.

### Role of circATP8A1 and related exosomes in gastric cancer confirmed by in vivo experiments

**Fig. 8:**
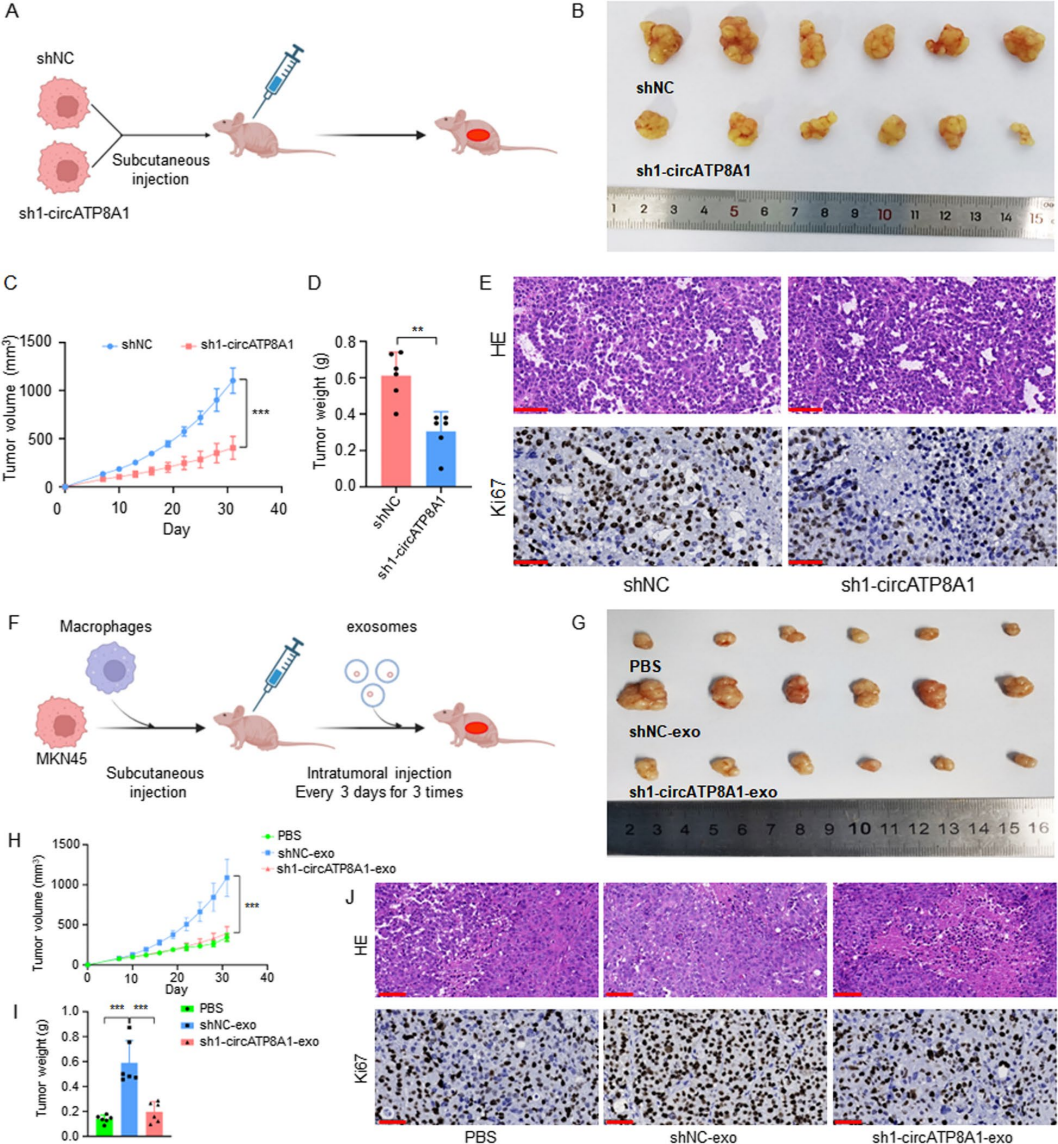
Role of circATP8A1 and related exosomes in gastric cancer confirmed by in vivo experiments. **A** Schematic diagram of cell transplantation of control group and circATP8A1 knockdown group. **B** Gross image indicates subcutaneous xenograft tumor model (*n* = 6). **C** Tumor growth curves of indicated groups were measured every 3 days for a total of 31 days. **D** Tumor weights were measured on day 31. **E** H&E staining and IHC staining of Ki-67 of circATP8A1 know-down group and control group. **F** Schematic diagram of cell transplantation and exosome injection. **G** Gross images of tissue specimens from the subcutaneous xenograft tumor model in nude mice. **H**-**I** Tumor growth rate and tumor weight of the subcutaneous xenograft tumor model in nude mice. **J** H&E staining and IHC staining of Ki-67 of indicate groups. * *P* < 0.05, ** *P* < 0.01, *** *P* < 0.001. *NS*, not significant. shNC, the knockdown-control group; sh1-circATP8A, circATP8A1 knockdown group1. OC-exo, exsomes from overexpression control cells; OE-exo, exosomes from cells overexpressing circATP8A1

To evaluate the oncogenic function of circATP8A1 in vivo, MKN-45 cells were stably transfected with circATP8A1 shRNA (Fig. 8A). A subcutaneous tumor model in nude mice demonstrates that the circATP8A1 knockdown group exhibited a significant reduction in tumor volume and tumor weight (Fig. 8B-D). HE staining shows pathology of the circATP8A1 knockdown group and the control group (Fig. 8E). Immunohistochemistry results showed that the protein expression of Ki67, a proliferation indicator, was decreased in the circATP8A1 knockdown group compared to the shNC control (Fig. 8E). These results suggest that the knockdown of circATP8A1 inhibits the progression of GC.

We further explored the function of exosomes in gastric cancer by mixing M0 macrophages with MKN-45 cells for tumor formation. Intratumoral injections of exosomes from MKN-45 cells knocking down circATP8A1 (sh1-circATP8A1-exo) and negative control (shNC-exo) (60ug/100ul/each) were given 3 times every 3 days after tumor formation (Fig. 8F). The results showed a significant increase in tumor volume and tumor weight in the shNC-exo group compared to the PBS-treated group. Tumor volume and tumor weight were significantly reduced in the sh1-circATP8A1-exo group compared to the shNC-exo group (Fig. 8G-I). HE staining shows pathology of the relative group (Fig. 8J). Immunohistochemistry results showed that Ki67 was increased in the shNC-exo group compared to the shNC control while the knockdown of circATP8A1 exosomes reversed the promoting effect (Fig. 8J). These results showed that exosomes from MKN-45 cells can promote tumor growth, and knockdown of circATP8A1 exosomes abolished the proliferation-promoting effect on gastric cancer cells.

## Discussion

Gastric cancer is the fifth most common cancer in the world and the third leading cause of cancer-related mortality worldwide [27]. However, the precise pathogenesis of gastric cancer remains unclear, and there is a lack of effective early diagnostic markers and therapeutic approaches.

The development of high-throughput sequencing technology has revealed an increasing number of circRNAs involved in human cancers. Previous research showed that circRNA exhibits extensive differential expression in gastric cancer. Chen et al. identified 180 differentially expressed circRNAs by RNA-seq analysis. Among them, 82 circRNAs were significantly up-regulated and 98 circRNAs were significantly down-regulated in gastric cancer tissues compared to normal gastric mucosa. Notably, approximately 80% of these circRNAs were generated by splicing of protein-coding genes [8]. Sui et al. identified a total of 467 differentially expressed circRNAs in gastric cancer using circRNA microarray data from 8 GC tissues and precancerous samples, of which 214 were significantly upregulated and 253 were significantly downregulated [28]. In this study, we screened differential circRNA expression profiles in gastric cancer by exosomes circRNA microarray assay and identified a novel circRNA circATP8A1 by tissue qPCR and plasma exosomes ddPCR. This circRNA originates from chromosome 4 and forms a loop by reverse splicing of exons 3 to 20 with a total length of 1558 bp. Its precursor is the mRNA for ATP8A1, hence given the name circATP8A1. This is a novel circRNA that has not yet been reported in the literature.

In this study, we found that circATP8A1 was highly expressed in gastric cancer tissues and plasma exosomes. Overexpression of circATP8A1 significantly promoted the proliferation, migration, and invasion of gastric cancer. Conversely, knockdown of circATP8A1 inhibited the proliferation, migration, and invasion of gastric cancer cells in vitro and in vivo. These results suggest that circATP8A1 is an oncogenic circRNA. Similar to our findings, Zhang J et al. found that circDLST promotes gastric carcinogenesis and metastasis by sponging adsorption of miR-502-5p and activation of the NRAS/MEK1/ERK1/2 signaling pathway [29]. CircNRIP1 acts as a miR-149-5p sponge to promote gastric cancer progression via the AKT1/mTOR pathway [9]. CircRNA CircCACTIN promotes gastric cancer progression by sponging MiR-331-3p and regulating TGFBR1 expression [30]. CiRS-7 is a well-known circRNA that can act as a miR-7 sponge to promote the progression of a variety of tumors. Han et al. found that ciRS-7 promotes gastric cancer progression by blocking the miR-7-mediated PTEN/PI3K/AKT pathway in GC cells [31]. circRNA may play an oncogenic role in gastric cancer.

Due to the insidious onset of gastric cancer, most cases are diagnosed at advanced stages. Thus, early detection is crucial for its treatment and prognosis. In our study, the RNAase digestion assay showed a significant reduction in linear ATP8A1, whereas the circRNA downregulated levels were not significant. The actinomycin D assay further demonstrated that circATP8A1 has a longer half-life compared to linear RNA. Due to their circular back-splicing structure, circRNAs can evade nuclease recognition, granting them a longer half-life and enhanced stability compared to linear RNA, allowing for prolonged presence in body fluids. Consistent with our results, previous studies have confirmed that Hsa_circ_0047905 and hsa_circ_0000096 can serve as reliable biomarkers for the diagnosis and prognosis of gastric cancer (AUC 0.85 and 0.82, respectively) [32, 33].

The tumor microenvironment contains a high number of immune cells, with a substantial proportion being tumor-associated macrophages. Exosomes are important mediators of intercellular information exchange. Our study confirmed that circATP8A1 is stably present in gastric cancer exosomes with significantly increased expression levels. Exosomes derived from SGC-7901 cells overexpressing circATP8A1 were able to promote macrophage M2 polarization and circATP8A1 expression was elevated in macrophages. Consistent with our findings, previous studies have shown that LncRNA RPPH1 promotes colorectal cancer metastasis by interacting with TUBB3 and promoting exosome-mediated macrophage M2 polarization [34]. Tumor-derived exosome miR-934 induces macrophage M2 polarization to promote liver metastasis in colorectal cancer [35]. Exosomes can promote tumor progression by promoting macrophage M2 polarization.

We further explored the mechanism by which the exosome circATP8A1 induced macrophage M2 polarization. STAT3 and STAT6 pathways are the core pathways for M2 polarization in macrophages [36]. In this study, we found that the STAT6 pathway was activated after exosome treatment of macrophages, revealing the important role of STAT6 in gastric cancer and M2 macrophages through single-cell sequencing and western blot. Previous studies demonstrated that IL-4 promotes macrophage M2 polarization mainly through activation of STAT6 [37]. M2 polarization in macrophages involves tyrosine phosphorylation and activation of STAT6, which further mediates the transcriptional activation of M2 macrophage-specific genes such as arginase 1 (Arg1) and mannose receptor 1 (Mrc1) [38]. M2-type markers were increased in macrophages overexpressing STAT6 [22], whereas STAT6 knockdown reduced M2-type marker expression [23], demonstrating the important role of STAT6 in macrophage M2 polarization. These results confirm that GC cells-derived exosome circATP8A1 promotes macrophage M2 polarization by affecting the STAT6 pathway.

The ceRNA mechanism is an important mechanism of circRNAs for biological functions. Cytoplasmic localization of circRNAs helps them interact with miRNA and play the role of miRNA sponge. In this study, we found that circATP8A1 was mainly distributed in the cytoplasm by FISH. The ceRNA networks were constructed by ENCORI. The biotin-labeled probe RNA pull-down experiment demonstrated the binding of miR-1-3p and circATP8A1. RIP experiments showed that both miR-1-3p and circATP8A1 could be pulled down by Ago2 antibody, confirming the existence of the RNA-induced silencing complex. The interaction between circATP8A1 and miR-1-3p was proved by the luciferin experiment. The upstream and downstream relationship of circATP8A1/miR-1-3p/STAT6 axis was further verified by rescue experiments. These results confirm that the gastric cancer cell-derived exosome circATP8A1 promotes macrophage M2 polarization by competitively binding miR-1-3p to activate the STAT6 pathway (Fig. 7G).

This study introduced several innovations. Firstly, we discovered and confirmed a previously unreported oncogenic circular RNA, circATP8A1, in gastric cancer plasma exosomes using circRNA microarray analysis. Secondly, we unveiled a novel mechanism through which circRNAs from gastric cancer exosomes induce macrophage polarization within the tumor microenvironment. Our research uniquely illustrated that exosomal circATP8A1 derived from gastric cancer activated the STAT6 pathway by competitively binding to miR-1-3p, consequently fostering M2 macrophage polarization and amplifying the progression of gastric cancer. In summary, our study screened and validated a novel oncogenic circRNA circATP8A1 which is upregulated in GC and associated with poorer prognosis. Single-cell sequencing data, WB, and immunofluorescence further confirmed the critical role of STAT6 in gastric cancer progression and macrophage M2 polarization. Using bioinformatics, qPCR, FISH, RIP assay, RNA pull-down assay, luciferase assay, and a rescue experiment, we verified a novel circATP8A1/miR-1-3p/STAT6 regulatory axis. Gastric cancer cell exosome circATP8A1 induces macrophage M2 polarization by competitively binding to miR-1-3p to activate the STAT6 pathway, which in turn promotes gastric cancer progression.

### Supplementary Information

**Additional file 1: Figure S1.** The fundamental characteristics of circATP8A1. **A** The genomic composition and circular structure of circATP8A1. **B** The expression of circATP8A1 in cell lines was verified by qPCR. **C** After treating AGS cells with Actinomycin D, the relative residual amounts of circATP8A1 and linear ATP8A1 were detected by qRT-PCR at different time points. CircATP8A1 (Circular circATP8A1), mRNA ATP8A1 (linear mRNA ATP8A1). **D** & **E** The relative residual amounts of circATP8A1 and linear ATP8A1 in AGS and MKN-45 cells before and after RNase treatment were detected by qRT-PCR. **F** Agarose gel electrophoresis was used to detect the expression of circATP8A1 (Circular circATP8A1) and ATP8A1 mRNA (linear mRNA ATP8A1) in the cDNA and gDNA, with β-actin serving as the control. *P* value is determined by t-test for B, D, and E. * *P* < 0.05, ** *P* < 0.01, *** *P* < 0.001. *NS*, not significant. **Figure S2.** CircATP8A1 knockdown reduces proliferation and migration in MKN-45 cells. **A** The relative expression levels of circATP8A1 and linear ATP8A1 after circATP8A1 knockout in MKN-45 cells were detected by qRT-PCR. **B** The proliferative ability of MKN-45 cells after circATP8A1 knockdown was detected by CCK8 assay. **C** & **D** Representative images of clone formation and statistics of colony counts in MKN-45 cells with circATP8A1 knockdown. **E** & **F** Microscopic images and quantification of the migration and invasion of MKN-45 cells as described above. *P* value is determined by t-test for A, B, D, and F. * *P* < 0.05, ** *P* < 0.01, *** *P* < 0.001. *NS*, not significant. shNC, the knockdown-control group; sh1-circATP8A, circATP8A1 knockdown group1; sh2-circATP8A, circATP8A1 knockdown group2. **Figure S3.** CircATP8A1 overexpression increases proliferation and migration in SGC7901 cells. **A** The relative expression levels of circATP8A1 and linear ATP8A1 after circATP8A1 overexpression in SGC-7901 cells were detected by qRT-PCR. **B** The proliferative ability of SGC-7901 cells after circATP8A1 overexpression was detected by CCK8 assay. **C** & **D** Representative images of clone formation and statistics of colony counts in SGC-7901 cells with circATP8A1 overexpression. **E** & **F** Microscopic images and quantification of the migration and invasion of SGC-7901 cells as described above (E & F). *P* value is determined by t-test for A, B, D, and F. * *P* < 0.05, ** *P* < 0.01, *** *P* < 0.001. *NS*, not significant. OC, overexpression control group; OE, circATP8A1 ovexpression group. **Figure S4.** Identification of exosomes from gastric cancer cell lines. **A** The exosomes of gastric cancer cell lines (AGS, SGC-7901, and MKN-45) were detected by electron microscopy. **B** Nanoparticle tracking analysis of exosomes in gastric cancer cell lines (AGS, SGC-7901, and MKN-45). **C** Western Blot analysis of exosome markers CD81, TSG101, and the negative marker Calnexin in gastric cancer cell lines. **Table S1.** The primers for qRT-PCR. **Table S2.** Association of circATP8A1 expression with clinicopathological features in plasma of gastric cancer patients.

## Data Availability

Supporting Information is available from the Wiley Online Library or the authors.
