# Supplementary material for: Exosome circATP8A1 induces macrophage M2 polarization by regulating the miR-1-3p/STAT6 axis to promote gastric cancer progression
Source: Mol Cancer. 2024 Mar 8;23:49. doi: 10.1186/s12943-024-01966-4 (PMC10921793; doi:10.1186/s12943-024-01966-4)
Supplement: Supplementary file 1 — Additional file 1: Figure S1. The fundamental characteristics of circATP8A1. A The genomic composition and circular structure of circATP8A1. B The expression of circATP8A1 in cell lines was verified by qPCR. C After treating AGS cells with Actinomycin D, the relative residual amounts of circATP8A1 and linear ATP8A1 were detected by qRT-PCR at different time points. CircATP8A1 (Circular circATP8A1), mRNA ATP8A1 (linear mRNA ATP8A1). D & E The relative residual amounts of circATP8A1 and linear ATP8A1 in AGS and MKN-45 cells before and after RNase treatment were detected by qRT-PCR. F Agarose gel electrophoresis was used to detect the expression of circATP8A1 (Circular circATP8A1) and ATP8A1 mRNA (linear mRNA ATP8A1) in the cDNA and gDNA, with β-actin serving as the control. P value is determined by t-test for B, D, and E. * P < 0.05, ** P < 0.01, *** P < 0.001. NS, not significant. Figure S2. CircATP8A1 knockdown reduces proliferation and migration in MKN-45 cells. A The relative expression levels of circATP8A1 and linear ATP8A1 after circATP8A1 knockout in MKN-45 cells were detected by qRT-PCR. B The proliferative ability of MKN-45 cells after circATP8A1 knockdown was detected by CCK8 assay. C & D Representative images of clone formation and statistics of colony counts in MKN-45 cells with circATP8A1 knockdown. E & F Microscopic images and quantification of the migration and invasion of MKN-45 cells as described above. P value is determined by t-test for A, B, D, and F. * P < 0.05, ** P < 0.01, *** P < 0.001. NS, not significant. shNC, the knockdown-control group; sh1-circATP8A, circATP8A1 knockdown group1; sh2-circATP8A, circATP8A1 knockdown group2. Figure S3. CircATP8A1 overexpression increases proliferation and migration in SGC7901 cells. A The relative expression levels of circATP8A1 and linear ATP8A1 after circATP8A1 overexpression in SGC-7901 cells were detected by qRT-PCR. B The proliferative ability of SGC-7901 cells after circATP8A1 overexpression was detect [file 12943_2024_1966_MOESM1_ESM.doc]

Exosome circATP8A1 induces macrophage M2 polarization through regulating the miR-1-3p/STAT6 axis to promote gastric cancer progression

Cuncan Deng^1#^, Mingyu Huo^1#^, Hongwu Chu^1#^, Xiaomei Zhuang^1#^, Guofei Deng^1^, Wenchao Li^1^, Hongfa Wei^1^, Leli Zeng^1^*, Yulong He^1^, Huashan Liu^2^, Jia Li^1^*, Changhua Zhang^1^*, Hengxing Chen^1^*.

**Supplemental Methods**

1. Plasma exosome circRNA microarray analysis

Plasma exosomes were obtained through ultracentrifugation of samples isolated from three healthy individuals and three patients with gastric cancer and processed by Heyuan Biotechnology for circRNA microarray analysis. Briely, Trizol RNA extraction reagent (Invitrogen) was used to isolate total RNA from the exosomes. Quantification was performed using the NanoDrop ND-2000, and the integrity of the RNA was assessed using the Agilent Bioanalyzer 2100. The purified total RNA underwent additional processing with the QIAGEN RNeasy Kit. Subsequently, the AffinityScript-RT kit, along with the Promoter Primer, was employed to perform reverse transcription on the isolated RNA, generating first-strand cDNA. The second strand of cDNA was synthesized using an Anti-sense Promoter. Finally, a T7 RNA polymerase reagent was added to facilitate cRNA amplification. The product was labeled with the fluorescent dye Cyanine-3-CTP (Cy3) and further purified using the QIAGEN RNeasy Kit.

Hybridization was performed at 65°C for 17 hours, and microarray slides were washed and scanned using the Agilent Scanner G5761A. Image processing and data extraction from the original images were performed using Feature Extraction software (Agilent Technologies, version 12.0.3.1). Finally, the raw data were subjected to analysis and processing using Genespring software (Agilent, version 14.8).

Following quantile normalization, the data underwent a filtering process. Subsequently, correlation coefficients were computed and visually represented for individual samples. Further analysis was conducted on the normalized data, with the criteria for selecting differential circRNAs in this study established as follows: a p-value less than 0.05 and a minimum fold change of 1.5. Statistical methods such as t-tests or ANOVA were employed to identify circRNAs that exhibited significant differences based on experimental type distinctions.

1. Actinomycin D assay

AGS cells were initially seeded in 6-well plates. After 24 hours, 2 ug/mL of actinomycin D was added into the culture, and subsequently, cells were collected at designated time intervals for qRT-PCR analysis. The resulting data were then normalized to the control group treated with the vehicle.

1. Detection of circRNA/miRNA/STAT6 expression, macrophage M1/M2 polarization markers and associated pathways using qPCR

THP1 cells were induced to differentiate into M0 macrophages through PMA stimulation. Subsequently, based on specific experimental conditions, various interventions were administered to the M0 macrophages. To assess their polarization, we utilized qPCR to detect markers associated with M1 (such as CD80 and CD86) and M2 (including CD163 and CD206) macrophages.

1. Immunohistochemical staining.

The gastric cancer tissue slides were obtained from the Pathology Departments of both the Seventh Affiliated Hospital and the First Affiliated Hospital of Sun Yat-sen University. Xylene and varying concentrations of alcohol were used for the deparaffinization and dehydration of all paraffin-embedded gastric cancer tissues. Antigen retrieval was then carried out using a citrate buffer and after washing with PBS, the slides were treated with a PBST solution containing 0.5% Triton-100 for 10-15 minutes at RT, and endogenous peroxidase activity was blocked by exposure to 3% H2O2 at RT for 30 minutes. A goat serum-blocking solution was applied at RT and allowed to incubate for 1 hour. Then, the primary antibody was added and incubated overnight at 4°C, followed by incubation with the corresponding biotinylated secondary antibody for 1 hour at RT. After each incubation step, the slides were thoroughly washed with PBS. The DAB reaction working solution was prepared at a 25:1 ratio, applied to the slides, and observed immediately under a microscope until the desired color development was achieved, at which point the reaction was stopped and hematoxylin working solution was added for 3 to 5 minutes. Slides were briefly immersed in a 1% hydrochloric acid alcohol solution for about 10 seconds, followed by rinsing with ddH2O. The slides were then sequentially placed in ethanol solutions with concentrations of 75%, 85%, 95%, and absolute ethanol, before being immersed in xylene and mounted.

(5) Kaplan-Meier plotter tool analysis

The Kaplan-Meier Plotter tool (http://kmplot.com/analysis/) was used to determine the association between STAT6 and the prognosis of gastric cancer patients.

(6) Western blot assay

Cellular proteins were extracted, separated on 10% SDS-PAGE gels, and subsequently transferred onto 0.22 μm PVDF membranes (Millipore, USA). Following this, the membranes were blocked using 5% skim milk powder and incubated with specific antibodies overnight at 4°C. The membranes were then exposed to the suitable secondary antibodies, and protein bands were visualized using an ECL detection system (Bio-Rad, USA). β-Actin served as the internal control.

(7) Single-cell sequencing data analysis of gastric cancer

Single-cell RNA-seq data were retrieved from the GEO database (GSE183904) and underwent a series of data processing steps, including quality control, data mapping, normalization, unsupervised clustering, and aggregated scoring.

To ensure data quality, three parameters, namely nFeature_RNA, nCount_RNA, and percent.MT, was employed for assessing cell quality, leading to the removal of cells with subpar quality. Following a quality assessment of read data, UMI counts were transformed into transcripts-per-10,000 using the Seurat package's "LogNormalize" method. Unsupervised clustering analyses were conducted on gene-expression matrices using the R package Seurat version 3.1.2. UMAPs and feature plots were generated using Seurat.

Additionally, an aggregated gene set score was calculated by employing the "AddModuleScore()" function based on the averaged expression of genes.

(8) Treatment of M0 macrophages with a STAT6 pathway inhibitor

Given the pivotal roles of STAT6 pathways in macrophage polarization, we conducted rescue experiments involving the STAT6 inhibitor AS1517499 (AS). THP1 cells were initially induced with PMA to promote their differentiation into M0 macrophages. Subsequently, these cells were exposed to either IL-4 alone or a combination of IL-4 and AS. After 48 hours, protein extraction was carried out to facilitate further analysis.

**Supplemental Figures**


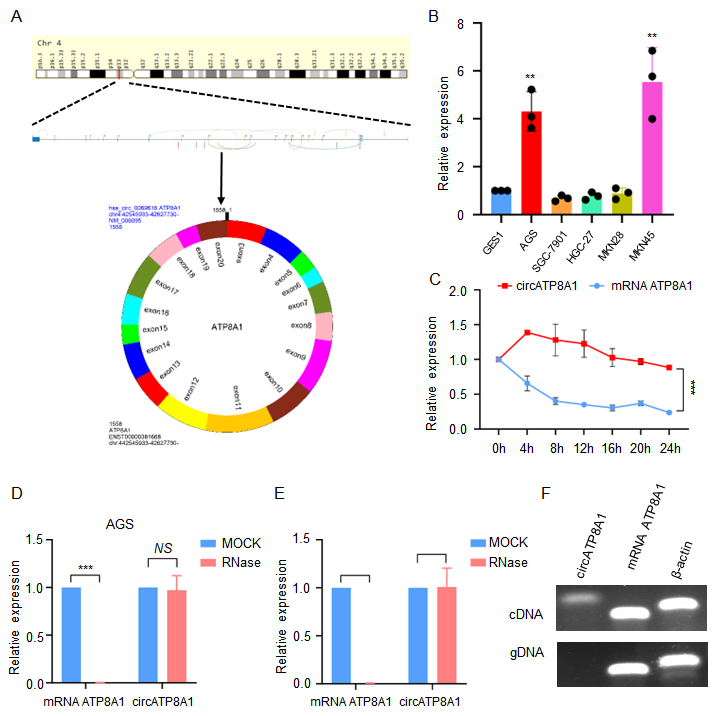


Figure S1. The fundamental characteristics of circATP8A1. A) The genomic composition and circular structure of circATP8A1. B) The expression of circATP8A1 in cell lines was verified by qPCR. C) After treating AGS cells with Actinomycin D, the relative residual amounts of circATP8A1 and linear ATP8A1 were detected by qRT-PCR at different time points. CircATP8A1 (Circular circATP8A1), mRNA ATP8A1 (linear circATP8A1). D & E) The relative residual amounts of circATP8A1 and linear ATP8A1 in AGS and MKN45 cells before and after RNase treatment were detected by qRT-PCR. F) Agarose gel electrophoresis was used to detect the expression of circATP8A1 (Circular circATP8A1) and ATP8A1 mRNA (linear circATP8A1) in the mRNA and DNA, with β-actin serving as the control. *P* value is determined by t-test for B, D and E.


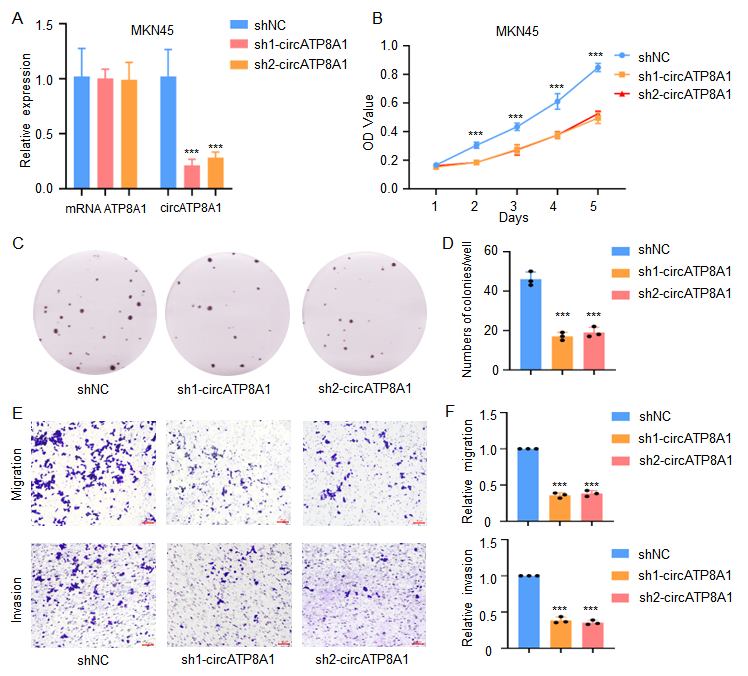


Figure S2. CircATP8A1 knockdown reduce proliferation and migration in MKN45 cells. A) The relative expression levels of circATP8A1 and linear ATP8A1 after circATP8A1 knockout in MKN45 cells were detected by qRT-PCR. B) The proliferative ability of MKN45 cells after circATP8A1 knockdown was detected by CCK8 assay. C & D) Representative images of clone formation and statistics of colony counts in MKN45 cells with circATP8A1 knockdown. E-F) Microscopic images and quantification of the migration and invasion of MKN45 cells as described above (E & F). P value is determined by t-test for A, B, D and F.


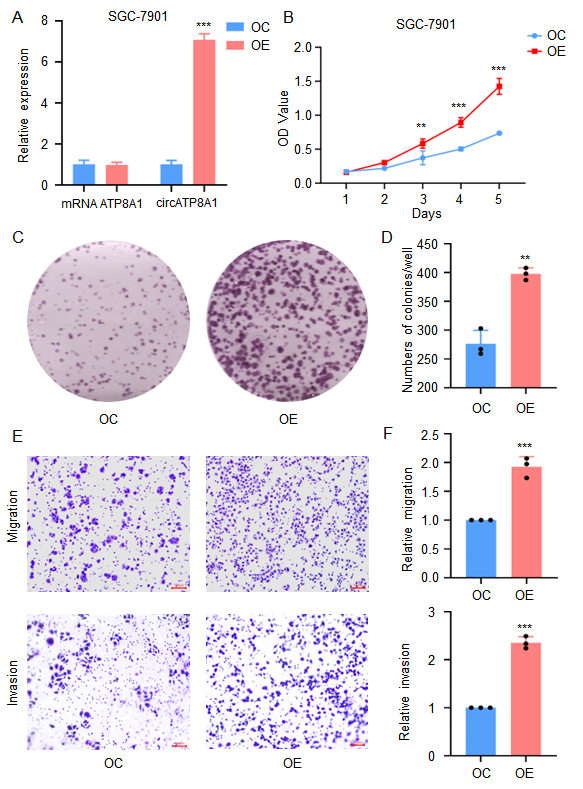


Figure S3. CircATP8A1 overexpression increase proliferation and migration in SGC7901 cells. A) The relative expression levels of circATP8A1 and linear ATP8A1 after circATP8A1 overexpression in SGC7901 cells were detected by qRT-PCR. B) The proliferative ability of SGC7901 cells after circATP8A1 overexpression was detected by CCK8 assay. C & D) Representative images of clone formation and statistics of colony counts in SGC7901 cells with circATP8A1 overexpression. E-F) Microscopic images and quantification of the migration and invasion of SGC7901 cells as described above (E & F). *P* value is determined by t-test for A, B, D and F.


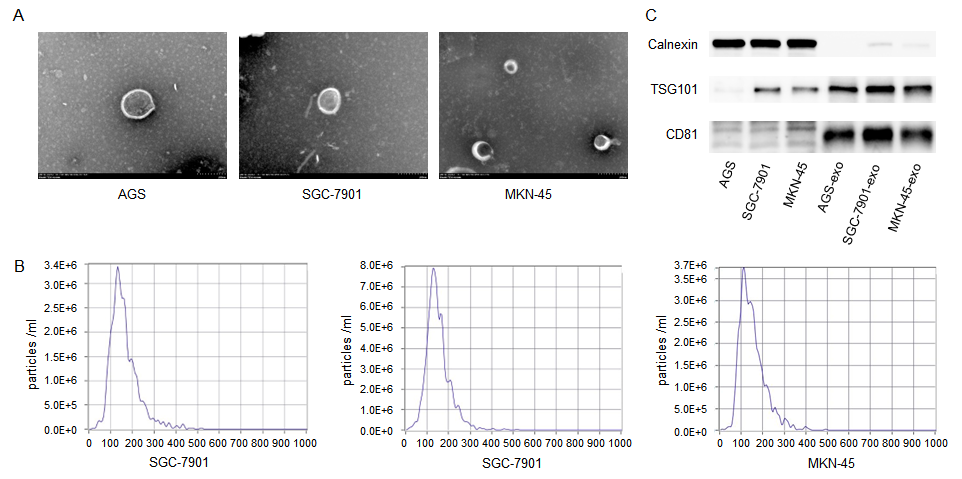


Figure S4. Identification of exosomes from gastric cancer cell lines. A) The exosomes of gastric cancer cell lines (AGS, SGC7901 and MKN45) were detected by electron microscopy. B) Nanoparticle tracking analysis of exosomes in gastric cancer cell lines (AGS, SGC7901 and MKN45). C) Western Blot analysis of exosome markers CD81, TSG101 and the negative marker Calnexin in gastric cancer cell lines.

Table S1. The primers for qRT-PCR

| Target | Forward primer | Reverse primer |
| --- | --- | --- |
| hsa_circ_0069616-1 | GAGGGAGCATTGGTCAGAGC | TCCCAGTGGACAATTTCCCA |
| hsa_circ_0069616-2 | GAGGGAGCATTGGTCAGAGC | TGTTGTATTTTGCAGTGAGCTCC |
| β-Actin | CACCATTGGCAATGAGCGGTTC | AGGTCTTTGCGGATGTCCACGT |
| ATP8A1 | ATATGTCAGGAGAGGCAGCC | GTACAACTGCTCCTGGGTCT |
| STAT6 | GATCAAGCGGTGTGAGCGGAAG | CAAGTGTGAAGCTGGCAGAGAAGAG |
| STAT3 | CACCAAGCGAGGACTGAGCATC | AGCCAGACCCAGAAGGAGAAGC |
| hsa-miR-1-3p-F | TGGAATGTAAAGAAGTATGTAT |  |
| hsa-miR-613-F | CGCAGGAATGTTCCTTCTTTGCC |  |
| hsa-miR-206-F | GCTGGAATGTAAGGAAGTGTGTGG |  |
| Random primer | NNNNNN |  |
| Oligo primer | TTTTTTTTTTTTTTTTTT |  |

Table S2. Association of circATP8A1 expression with clinicopathological features in plasma of gastric cancer patients.

| **Characteristics**  **(*n*=36)** | **CircATP8A1 expression** | | |
| --- | --- | --- | --- |
|  | **Negative(*n*=18)** | **Positive(*n*=18)** | ***P*** |
| Age (years) |  |  | 0.505 |
| <60 | 10 | 7 |  |
| ≥60 | 8 | 11 |  |
| Gender |  |  | 0.733 |
| Male | 10 | 12 |  |
| Female | 8 | 6 |  |
| Weight | 56.89±12.15 | 56.28±11.21 | 0.972 |
| Height | 161.39±7.71 | 160.39±7.24 | 0.593 |
| Location |  |  | 0.924 |
| Upper third | 5 | 6 |  |
| Middle third | 5 | 5 |  |
| Lower third | 8 | 7 |  |
| Whole | 0 | 0 |  |
| T stage |  |  | 0.653 |
| T1 | 5 | 2 |  |
| T2 | 1 | 1 |  |
| T3 | 3 | 4 |  |
| T4 | 9 | 11 |  |
| N stage |  |  | 0.463 |
| N0 | 9 | 5 |  |
| N1 | 3 | 4 |  |
| N2 | 2 | 5 |  |
| N3 | 4 | 4 |  |
| M stage |  |  | 0.005 |
| Negative | 16 | 7 |  |
| Positive | 2 | 11 |  |
| TNM Stage |  |  | 0.325 |
| Ⅰ | 6 | 2 |  |
| Ⅱ | 5 | 4 |  |
| Ⅲ | 5 | 8 |  |
| Ⅳ | 2 | 4 |  |
| Differentiation |  |  | 0.289 |
| Well | 0 | 0 |  |
| Moderately | 8 | 4 |  |
| Poorly | 10 | 14 |  |
